# Durable Intracranial Response to Sacituzumab Tirumotecan in HER2‐Negative Salivary Duct Carcinoma With High TROP2 Expression: A Case Report

**DOI:** 10.1002/cnr2.70676

**Published:** 2026-09-21

**Authors:** Guang‐Liang Chen, Biao Tan, Sufen Cao, Xin Liu, Yanjing Guo, Dongmei Ji

**Affiliations:** ^1^ Department of Medical Oncology Fudan University Shanghai Cancer Center Shanghai China; ^2^ Department of Oncology, Shanghai Medical College Fudan University Shanghai China; ^3^ Department of Medical Oncology Lichuan Ethnic Hospital of Traditional Chinese Medicine Lichuan Hubei China; ^4^ Department of Nursing Fudan University Shanghai Cancer Center Shanghai China

**Keywords:** antibody‐drug conjugate, case report, CNS metastasis, sacituzumab tirumotecan, salivary duct carcinoma, TROP2

## Abstract

**Background:**

Salivary duct carcinoma is an aggressive salivary gland malignancy in which systemic therapy is often guided by androgen receptor and HER2 status. Treatment options are limited for HER2‐negative disease after progression on androgen‐deprivation therapy and chemotherapy, particularly when active central nervous system (CNS) involvement develops.

**Case:**

A 62‐year‐old man with androgen receptor‐positive, HER2‐negative salivary duct carcinoma developed progressive intracranial dural metastases involving the left temporal and occipital regions after surgery, adjuvant radiotherapy, androgen‐deprivation therapy, and multiple chemotherapy‐based regimens. The dominant lesion was a 33.6‐mm left temporal dural‐based mass extending across the left tentorial leaflet. Immunohistochemical profiling of archival primary tumor tissue showed high membranous TROP2 expression (H‐score, 230). Off‐label sacituzumab tirumotecan was initiated at 350 mg every 2 weeks without concurrent corticosteroids or local CNS‐directed therapy. Serial magnetic resonance imaging demonstrated sustained regression of the dominant left temporal mass from 33.6 to 15.3 mm (−54.5%), with no new intracranial lesions. Intracranial disease control was maintained for approximately 9 months. Treatment‐related Grade 2 oral mucositis and leukopenia improved with supportive care and did not require dose reduction or treatment discontinuation.

**Conclusion:**

In heavily pretreated, HER2‐negative salivary duct carcinoma with high TROP2 expression and progressive intracranial dural metastases, sacituzumab tirumotecan was associated with a durable intracranial response without local CNS‐directed therapy. This observation supports further clinical evaluation of TROP2‐directed antibody–drug conjugates in salivary duct carcinoma with CNS involvement, while the predictive value of TROP2 expression remains to be established.

## 1 Introduction

Salivary duct carcinoma (SDC) is a rare and aggressive salivary gland malignancy with morphologic and biologic overlap with high‐grade invasive ductal carcinoma of the breast [1, 2]. SDC carries a high risk of locoregional recurrence and distant metastasis [3]. Many tumors express androgen receptor (AR), and a subset shows HER2 amplification or overexpression, supporting biomarker‐guided treatment with androgen deprivation therapy or HER2‐directed therapy [4]. However, therapeutic options remain limited for patients with HER2‐negative disease after progression on AR‐directed therapy and chemotherapy [2, 5].

Central nervous system (CNS) involvement is a clinically important complication of SDC. In the largest multicenter retrospective study, brain metastases were identified in 65 of 464 patients with SDC (14%) [6]. Once CNS involvement develops, treatment is challenging because available evidence is derived mainly from retrospective series and case reports, and local therapy remains central to management [2, 7, 8, 9]. Effective systemic treatment for active intracranial disease in SDC remains poorly established, particularly in patients with HER2‐negative tumors for whom HER2‐directed therapies are not applicable.

Antibody–drug conjugates (ADCs) have expanded treatment options in several refractory epithelial cancers [10]. Trophoblast cell surface antigen 2 (TROP2) is the target of approved and investigational ADCs across multiple solid tumors, and TROP2 expression has been reported in salivary gland carcinomas, including SDC [10, 11, 12]. Preliminary CNS activity of TROP2‐directed ADCs in SGC has also emerged. In a 2025 master‐protocol study of ESG401, all three patients with brain metastases achieved an intracranial partial or complete response, although histology‐specific outcomes and response durability were not reported [13]. Here, we describe a patient with HER2‐negative SDC, high TROP2 expression in archival primary tumor tissue, and progressive dural‐based intracranial disease who experienced a durable radiographic response during sacituzumab tirumotecan treatment without concurrent corticosteroids or local CNS‐directed therapy.

## 2 Case Presentation

### 2.1 Patient Information and Initial Management

**FIGURE 1:**
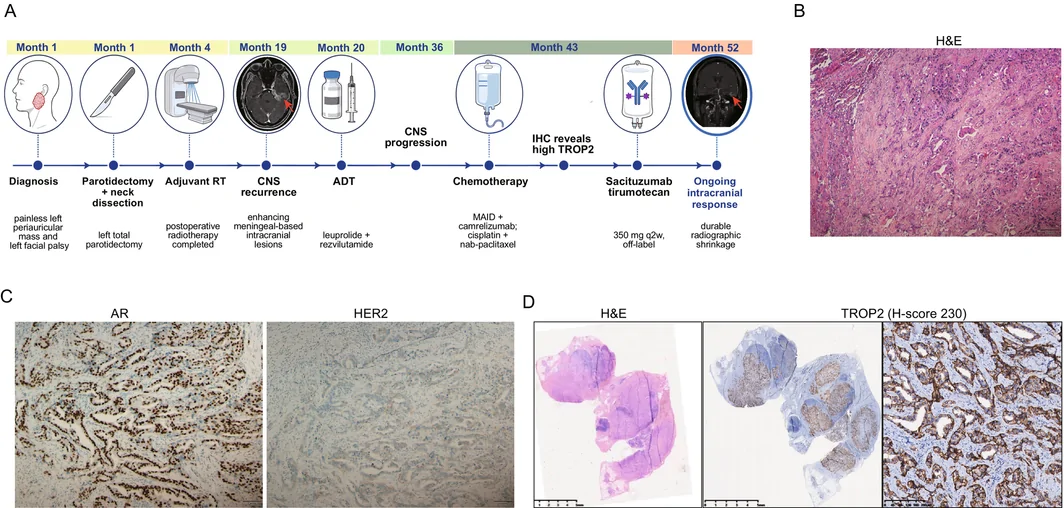
Clinical course and pathologic features. (A) Clinical timeline showing diagnosis, surgery, adjuvant radiotherapy, CNS recurrence, androgen‐deprivation therapy, chemotherapy‐containing regimens, TROP2 testing, and sacituzumab tirumotecan initiation. Clinical events are presented as relative months, with Month 1 corresponding to the initial clinical presentation in January 2022. (B) Hematoxylin and eosin staining showed poorly differentiated salivary duct carcinoma. (C) Immunohistochemistry demonstrated androgen receptor positivity and HER2 IHC 0. (D) Exploratory immunohistochemistry of archival primary tumor tissue showed strong membranous TROP2 expression (H‐score, 230). Abbreviations: AR, androgen receptor; CNS, central nervous system; HER2, human epidermal growth factor receptor 2; IHC, immunohistochemistry; TROP2, trophoblast cell‐surface antigen 2.

In January 2022, a 62‐year‐old man presented to Fudan University Shanghai Cancer Center with a painless left periauricular mass and left‐sided facial palsy (Figure 1A). Initial PET/CT demonstrated a hypermetabolic left parotid lesion and ipsilateral upper cervical lymphadenopathy, without evidence of distant metastatic disease. He underwent left total parotidectomy and neck dissection. Histopathologic examination showed poorly differentiated SDC measuring 1.7 cm, with perineural invasion (Figure 1B). Regional nodal metastases were present in 3 of 19 left supraomohyoid lymph nodes; the largest metastatic node measured 1.0 cm, without extranodal extension. Based on the preoperative facial nerve palsy, multiple ipsilateral nodal metastases, and absence of distant disease, the tumor was staged as T4aN2bM0, stage IVA. Immunohistochemistry showed expression of CK7, AR, vimentin, and GCDFP‐15, with a Ki‐67 proliferation index of approximately 50% (Figure 1C and Table S1). HER2 expression was absent (IHC 0) (Figure 1C). Targeted sequencing identified a pathogenic TP53 truncating variant (c.916C>T, p.Arg306Ter), and fluorescence in situ hybridization showed no ETV6 rearrangement.

### 2.2 Disease Course and Therapeutic Interventions

The patient completed adjuvant radiotherapy by Month 4. At Month 19, routine surveillance MRI of the parotid region incidentally revealed intracranial abnormalities within the field of view. This finding prompted dedicated contrast‐enhanced brain MRI, which showed enhancing dural‐based intracranial lesions consistent with CNS metastatic recurrence. Biopsy of the intracranial lesions was not performed because the patient declined the procedure. Combined androgen‐deprivation therapy with leuprolide and rezvilutamide was started at Month 20 and achieved transient disease stabilization. At Month 36, brain MRI showed progression of plaque‐like dural thickening and mass‐forming lesions in the left temporal and occipital regions, with increased vasogenic edema. Symptoms also worsened, particularly persistent pressure‐like pain involving the left periauricular, temporal, and occipital regions. Given the rapid intracranial progression and lack of established systemic options, the patient received MAID plus camrelizumab as an empiric salvage regimen following multidisciplinary discussion. MAID comprised mesna, doxorubicin, ifosfamide, and dacarbazine. After further intracranial progression, treatment was changed to cisplatin plus nab‐paclitaxel. Despite these treatments, the intracranial disease continued to progress.

**FIGURE 2:**
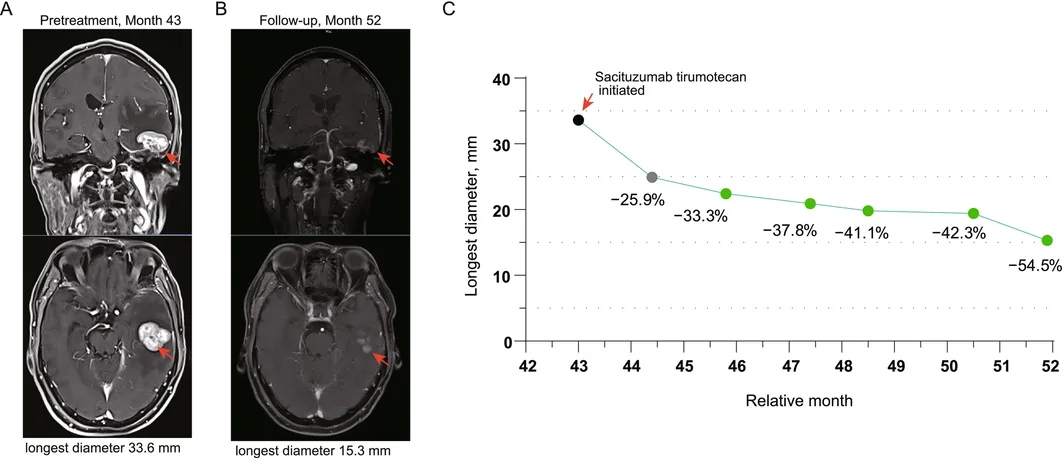
Sustained radiographic regression of a dural‐based intracranial metastasis during sacituzumab tirumotecan treatment. (A) Pretreatment contrast‐enhanced brain MRI at Month 43 showed a measurable dural‐based extra‐axial mass centered in the left temporal region, straddling the left tentorial leaflet and involving the left sphenoid wing. The longest diameter was 33.6 mm. (B) Follow‐up MRI at Month 52 showed reduction of the lesion to 15.3 mm, representing a 54.5% decrease from baseline. (C) Serial measurements of the lesion's longest diameter, plotted by relative month from the initial clinical presentation, demonstrated sustained tumor regression during sacituzumab tirumotecan treatment. Treatment was initiated at Month 43, and point labels indicate percentage change from the pretreatment baseline.

Exploratory immunohistochemical analysis of archival primary tumor tissue showed strong membranous TROP2 expression, with an H‐score of 230 (Figure 1D). Before sacituzumab tirumotecan initiation, contrast‐enhanced brain MRI at Month 43 demonstrated a measurable dural‐based mass centered in the left temporal region, straddling the left tentorial leaflet and involving the left sphenoid wing. The mass measured 33.6 mm in longest diameter (Figure 2). After discussion of the limited clinical evidence, potential risks, expected benefits, and available alternatives, sacituzumab tirumotecan was initiated off‐label at 350 mg every 2 weeks. No corticosteroids, surgery, stereotactic radiosurgery, whole‐brain radiotherapy, or other local CNS‐directed therapy was administered during response assessment.

### 2.3 Follow‐Up and Outcomes

Serial MRI showed sustained intracranial tumor shrinkage. By Month 52, the target lesion had decreased from 33.6 to 15.3 mm, representing a 54.5% reduction from baseline. No new intracranial lesions were identified, and the non‐target lesions showed no unequivocal progression. Peritumoral edema and neurologic symptoms improved, and the patient remained on treatment at the last follow‐up. Adverse events were graded according to the Common Terminology Criteria for Adverse Events, version 5.0. Grade 2 oral mucositis and leukopenia occurred; both improved with supportive care without requiring dose reduction or treatment discontinuation.

## 3 Discussion

This case suggests intracranial activity of sacituzumab tirumotecan in heavily pretreated, HER2‐negative SDC with high TROP2 expression and progressive dural metastases. The dominant left temporal lesion decreased by 54.5%, and intracranial disease control was maintained through approximately 9 months of follow‐up. Before sacituzumab tirumotecan initiation, the CNS disease had continued to progress despite androgen‐deprivation therapy and two chemotherapy‐containing regimens. The response occurred without concurrent corticosteroids or local CNS‐directed therapy. In addition, adjuvant radiotherapy had been completed 39 months earlier, making a delayed radiation effect unlikely. Although a single case cannot establish efficacy or validate TROP2 expression as a predictive biomarker, the observed response supports further evaluation of TROP2‐directed ADCs in CNS‐involved, HER2‐negative SDC, a setting in which effective systemic treatment remains undefined [2].

Evidence guiding the treatment of CNS‐involved SDC is limited to retrospective series and case reports [7, 8, 9, 14]. Most reported patients received surgery, radiotherapy, or both. Systemic responses have been described mainly in AR‐positive or HER2‐positive disease treated with androgen‐deprivation or HER2‐directed therapy (Table 1) [6, 7, 8, 9, 15, 16, 17, 18]. The present case extends this limited clinical experience to a TROP2‐directed ADC in HER2‐negative SDC.

**TABLE 1:** Selected reports of CNS involvement in salivary duct carcinoma with treatment information.

Study	CNS pattern	Molecular context	Treatment	Outcome
Sorenson et al. [7]	Brain metastasis	HER2‐positive	Neratinib	Transient clinical response/CNS stabilization
Rösch et al. [8]	Intracranial/extra‐axial metastases	AR‐positive; HER2 IHC 2+ no amplification	ADT with goserelin after RT and trastuzumab failure	Very good partial remission
Gazola et al. [9]	CNS metastasis	HER2‐positive	Fam‐trastuzumab deruxtecan	Excellent response
Kishi et al. [15]	Left frontal brain metastasis	HER2‐directed treatment context	Resection + cavity RT; paclitaxel + trastuzumab	No recurrence at 18 months
Zhu et al. [16]	Cranial nerve lesions + intracranial metastasis	AR positive; HER2 IHC 2+	Local therapy	Limited response data
Zheng et al. (ASCO Abstract 6100) [13]	CNS involvement reported; anatomy unspecified	TROP2‐positive SGC; histology unspecified	ESG401	Preliminary activity; CNS‐specific outcomes unavailable
Present case	Measurable dural‐based, extra‐axial disease	HER2‐negative SDC; high TROP2 expression	Sacituzumab tirumotecan; no steroids/local CNS therapy	54.5% tumor reduction; intracranial control for ~9 months

*Note:* The table summarizes patient‐level reports of CNS‐involved SDC and relevant evidence for TROP2‐directed ADCs in SGC. Aggregate database studies and non‐SGC tumors were excluded.

Abbreviations: ADC, antibody–drug conjugate; ADT, androgen‐deprivation therapy; AR, androgen receptor; CNS, central nervous system; HER2, human epidermal growth factor receptor 2; IHC, immunohistochemistry; RT, radiotherapy; SDC, salivary duct carcinoma; SGC, salivary gland carcinoma; TROP2, trophoblast cell‐surface antigen 2.

TROP2 expression in the archival primary tumor provided a biological rationale for sacituzumab tirumotecan treatment [11]. TROP2 is expressed in a subset of salivary gland carcinomas, including SDC, and has been investigated as a therapeutic target for ADCs [11, 19]. However, the responding CNS lesion was not sampled, and its TROP2 concordance with the primary tumor remains unknown. A small paired analysis across solid tumors reported 78.6% concordance in TROP2 expression between primary tumors and brain metastases, indicating incomplete cross‐site concordance; comparable data are unavailable for SDC [20]. Thus, this case cannot establish TROP2 expression as a predictive biomarker of treatment benefit or determine its treatment‐independent prognostic significance.

Clinical evidence for TROP2‐directed ADCs in salivary gland carcinoma remains preliminary. Early ESG401 data suggest activity in TROP2‐positive disease, including patients with CNS involvement [13]. However, the aggregate results do not allow assessment by histologic subtype, HER2 or AR status, CNS anatomy, concurrent treatment, or response durability. Sacituzumab tirumotecan is also being evaluated prospectively in triple‐negative breast cancer with active brain metastases, including leptomeningeal disease [10]. These emerging data support further investigation but do not establish intracranial efficacy in HER2‐negative SDC.

Interpretation of this case is limited by its single‐patient design, the absence of pharmacokinetic or tissue‐distribution data, and the dural‐based, extra‐axial location of the responding lesion. Drug exposure may differ among dural, parenchymal, and leptomeningeal compartments; therefore, the observed response should not be generalized to other patterns of CNS involvement. Future studies should incorporate standardized TROP2 assessment, paired analysis of primary and metastatic lesions, and predefined CNS response criteria. They should also determine whether TROP2 expression modifies treatment benefit rather than merely confirming target presence.

## 4 Conclusions

The observed response provides preliminary support for further investigation of sacituzumab tirumotecan and other TROP2‐directed ADCs in CNS‐involved, HER2‐negative SDC. Future studies should determine whether activity extends to parenchymal and leptomeningeal disease and whether TROP2 expression can guide patient selection.

## Author Contributions

**Guang‐Liang Chen:** investigation, funding acquisition, writing – original draft, writing – review and editing, visualization, formal analysis, data curation, resources, project administration, validation. **Biao Tan:** investigation, writing – original draft, writing – review and editing, visualization, validation, formal analysis, project administration, resources, data curation. **Sufen Cao:** investigation, validation, visualization, data curation, resources. **Xin Liu:** resources, validation, writing – original draft, writing – review and editing. **Yanjing Guo:** investigation, visualization, validation, resources, formal analysis, data curation, writing – original draft, writing – review and editing. **Dongmei Ji:** conceptualization, investigation, resources, supervision, data curation, formal analysis, visualization, validation.

## Funding

This work was supported by National Natural Science Foundation of China (82573874).

## Ethics Statement

The Ethics Committee of Fudan University Shanghai Cancer Center granted an exemption from formal ethical review for this single‐patient case report (2607‐Exemption‐No003). Consent to participate was not applicable.

## Consent

The patient provided written informed consent for publication of the clinical information and accompanying images.

## Conflicts of Interest

The authors declare no conflicts of interest.

## Supporting information


**Table S1:** Full immunohistochemical and molecular profile.

## Data Availability

The data supporting the findings of this case report are available from the corresponding author upon reasonable request, subject to applicable patient privacy and ethical restrictions.
