# Supplementary material for: Durable Intracranial Response to Sacituzumab Tirumotecan in HER2‐Negative Salivary Duct Carcinoma With High TROP2 Expression: A Case Report
Source: Cancer Rep (Hoboken). 2026 Sep 21;9(9):e70676. doi: 10.1002/cnr2.70676 (PMC13593920; doi:10.1002/cnr2.70676)
Supplement: Supplementary file 1 [file CNR2-9-e70676-s001.docx]

**Supplementary Tables**

**Table S1. Full immunohistochemical and molecular profile**

| **Marker / Test** | **Result** |
| --- | --- |
| Archival primary tumor | |
| CK7 | Positive |
| AR | Positive; nuclear expression in 85% of tumor cells |
| Vimentin | Positive |
| GCDFP-15 | Positive |
| S100 | Negative |
| P63 | Negative |
| HER2 | IHC 0 |
| Ki-67 | ~50% |
| TROP2 | High membranous expression, H-score 230 |
| EGFR | H-score 220 |
| c-MET | H-score 37 |
| B7-H3 | H-score 16 |
| Targeted next-generation sequencing | |
| TP53 | c.916C>T, p.Arg306Ter |
| RET | Wild-type |
| HRAS | Wild-type |
| PIK3CA | Wild-type |
| BRAF | Wild-type |
| PTEN | Wild-type |
| ERBB2 amplification | Not detected |
| NTRK | Not detected |
| ETV6 FISH | No rearrangement detected |
